# Editorial: Ovarian aging: pathophysiology and recent development of maintaining ovarian reserve, volume IV

**DOI:** 10.3389/fendo.2026.1888968

**Published:** 2026-06-11

**Authors:** Osamu Wada-Hiraike, Michio Kitajima, Antonio Simone Laganà

**Affiliations:** 1 Department of Obstetrics and Gynecology, Nippon Medical School, Bunkyo, Japan; 2 Women's Medical Center, Takagi Hospital/Department of Obstetrics and Gynecology, School of Medicine International University of Health and Welfare, Fukuoka, Japan; 3 Unit of Obstetrics and Gynecology, "Paolo Giaccone" Hospital, Department of Health Promotion, Mother and Child Care, Internal Medicine and Medical Specialties (PROMISE), University of Palermo, Palermo, Italy

**Keywords:** assisted reproductive technology, diminished ovarian reserve, fertility preservation, oocyte quality, ovarian aging, ovarian reserve, oxidative stress, primary ovarian insufficiency

## Introduction

To counteract ovarian aging is one of the central challenges in reproductive medicine. The gradual depletion of the follicular pool and the accompanying deterioration in oocyte quality are inevitable events that underlie the age-related decline in female fertility. Although assisted reproductive technology has expanded reproductive options, it cannot fully overcome the effects of ovarian aging and primary ovarian insufficiency (POI). Therefore, understanding the mechanisms of ovarian aging and developing strategies to maintain ovarian reserve remain urgent priorities.

This Research Topic was organized to provide updated insights into the pathophysiology of ovarian aging and recent developments aimed at preserving or restoring ovarian function. Owing to the contribution of researchers, this topic is comprised of a fourth volume and 17 excellent articles are collected here covering a wide range of subjects, including follicular depletion, mitochondrial dysfunction, oxidative stress, inflammatory biomarkers, mathematical modeling of reproductive outcomes, endocrine and regenerative therapies, complementary medicine, fertility preservation, and clinical outcomes in women with diminished ovarian reserve or POI. Together, these articles illustrate the multidisciplinary nature of current ovarian aging research, linking basic science, translational medicine, reproductive endocrinology, assisted reproduction, and women’s health across the lifespan.

Several contributions addressed the biological framework of ovarian aging. Hirano et al. reviewed the pathophysiology of ovarian aging and recent developments in maintaining ovarian reserve. They emphasized that ovarian aging is characterized not only by a quantitative decline in the primordial follicle pool but also by deterioration in follicle and oocyte quality, involving genetic predisposition, hormonal changes, mitochondrial dysfunction, oxidative stress, and alterations in the ovarian microenvironment. They also summarized emerging strategies such as antioxidant and mitochondria-targeted therapies, hormonal modulation, growth factor interventions, mitochondrial transfer, *in vitro* follicle activation, ovarian tissue cryopreservation, and pharmacological inhibition of follicle depletion. Muhammad further broadened this perspective by framing reproductive aging as a systemic neuroendocrine, immune, metabolic, and mitochondrial transition. This review highlighted the importance of the hypothalamic-pituitary-gonadal axis, kisspeptin and neurokinin B signaling, estrogen receptor biology, inflammation, oxidative stress, and environmental and genetic modifiers in shaping the menopausal transition.

The ovarian microenvironment was explored in depth by Wang et al., who used proximity extension assay technology to identify inflammatory biomarkers in serum and follicular fluid. Their prospective study also suggested that oncostatin M and related inflammatory pathways, including IL-10 anti-inflammatory signaling and PI3K-Akt signaling, may be associated with follicular development and ovarian reserve. These findings support the emerging concept that ovarian reserve is influenced not only by follicle number but also by the inflammatory and paracrine environment surrounding the oocyte. Lu et al. examined another mechanistic aspect of ovarian damage by investigating whether loss of p21 protects against POI induced by alkylating agents. Their findings showed that p21 deficiency did not prevent chemotherapy-induced ovarian insufficiency in mice, providing an important negative result and suggesting that cellular senescence pathways in ovarian injury are more complex than single-molecule targeting.

Several articles examined how ovarian reserve and oocyte quality translate into artificial reproductive technology (ART) outcomes. Sujino et al. developed a comprehensive mathematical model integrating age-dependent oocyte quantity and quality to predict live birth rate. Their model incorporated clinical IVF data, oocyte quality, ovarian reserve markers, and logistic regression, and demonstrated high predictive performance. Such work is particularly valuable because reproductive counseling increasingly requires individualized prediction rather than reliance on chronological age alone. Carnesi et al. reported that diminished ovarian reserve was associated with embryo euploidy rate in a single-center study of patients undergoing IVF and PGT-A, suggesting that ovarian reserve markers such as AMH and AFC may reflect not only the quantity but also aspects of oocyte or embryo quality. Sun et al. analyzed poor ovarian responders undergoing IVF/ICSI and identified maternal age and progesterone level on the trigger day as independent predictors of clinical pregnancy. Liu et al. studied young women with diminished ovarian reserve undergoing frozen embryo transfer and examined pregnancy outcomes together with exploratory insights into endometrial aging. These studies collectively reinforce that ovarian aging affects ART through multiple pathways, including ovarian response, oocyte competence, embryo chromosomal status, endocrine dynamics, and potentially endometrial receptivity.

Growth hormone was the focus of two reviews. Han et al. reviewed the role of growth hormone in ART for patients with diminished ovarian reserve, discussing its potential effects on oxidative stress, follicular development, oocyte quality, and endometrial receptivity. Yang et al. provided a complementary review of growth hormone regulation of ovarian function, with particular attention to mechanisms, formulations, and therapeutic applications. They highlighted novel biomaterial-based delivery systems designed to improve growth hormone bioavailability, targeting, and therapeutic efficiency. Although growth hormone remains a promising adjuvant in selected patients with diminished ovarian reserve or poor ovarian response, these reviews also indicate that clinical application requires careful patient selection, standardized protocols, and stronger evidence regarding efficacy and safety.

Oxidative stress was another recurring theme. Xie et al. [11] reviewed antioxidant strategies for preserving female reproductive capacity in the context of ovarian aging and cryopreservation. They described how reactive oxygen species impair mitochondrial function, DNA integrity, spindle assembly, chromosome cohesion, and embryo competence, and how cryopreservation itself can induce oxidative injury, apoptosis, and follicular loss. This article links ovarian aging research directly to fertility preservation, particularly for women facing age-related decline or gonadotoxic treatment.

Regenerative medicine was represented by Merhi et al., who systematically reviewed endocrine and regenerative mechanisms of adipose-derived stem cells in female infertility. Their review summarized preclinical and early clinical evidence suggesting that adipose-derived stem cells and their derivatives may improve endometrial thickness and receptivity, partially restore ovarian function in POI or chemotherapy-induced damage, reduce oxidative stress, and support oocyte and embryo development. However, the authors also emphasized that most evidence remains preclinical or based on small early-phase studies, making standardized protocols and long-term safety evaluation essential before clinical integration.

Complementary and traditional medicine approaches formed another important part of this collection. In this regard, POI and diminished ovarian reserve were also addressed from therapeutic and long-term health perspectives. Fu et al. [13] conducted a randomized controlled trial of KunTai capsules which contain natural products, combined with Femoston (a bioidentical product containing 17 beta estradiol and a progestogen, usually dydrogesterone) in women with premature ovarian insufficiency and reported greater improvement in lumbar spine bone mineral density and menopausal symptoms compared with use of Femoston alone. This study is notable because ovarian insufficiency has consequences beyond infertility, including hypoestrogenic symptoms and skeletal health. Of note, Femoston is approved for use in Europe, Australia and many other international markets, but is not approved for use in the U.S. or Canada. Yang et al. reviewed non-hormone replacement approaches to POI, focusing on natural products and stem cells targeting autophagy. This review emphasized autophagy as a potential mechanistic target in oocyte development, follicular survival, and ovarian reserve maintenance. He et al. [15] reviewed the potential impact of electroacupuncture on POI, summarizing possible mechanisms involving regulation of the hypothalamic-pituitary-ovarian axis, neuroendocrine-immune balance, inflammation, ovarian blood flow, mesenchymal stem cell activity, gut microbiota, apoptosis, oxidative stress, and PI3K/Akt and Hippo signaling. Liu et al. [16] presented a multicenter randomized controlled trial protocol evaluating acupuncture pretreatment in women with diminished ovarian reserve undergoing IVF-ET. This planned trial will randomize 300 women to acupuncture or placebo acupuncture, with clinical pregnancy rate after first embryo transfer as the primary endpoint. These contributions are valuable because they move complementary medicine toward more rigorous mechanistic evaluation and controlled clinical testing.

Finally, Min et al. provided a systematic scoping review of rodent models of functional hypothalamic amenorrhea. Although functional hypothalamic amenorrhea is distinct from intrinsic ovarian aging, it shares clinically relevant features such as menstrual disturbance, hypoestrogenism, infertility, and long-term skeletal consequences. Their review highlighted the heterogeneity of animal models, including differences in dietary and exercise interventions, estrous cycle monitoring, and mechanistic endpoints. This article underscores the importance of robust and standardized preclinical models for studying reproductive endocrine disorders and for translating mechanistic insights into possible clinical interventions.

## Concluding remarks

Taken together, these articles demonstrate that ovarian aging is not a single biological event but a multidimensional process. The Research Topic also shows that ovarian reserve should not be evaluated solely by chronological age or a single biomarker. Instead, it requires integrated assessment of ovarian reserve markers, follicular biology, embryo competence, endocrine status, ART outcomes, and long-term women’s health. This Research Topic also highlights several directions for future research. However, many potentially promising interventions remain insufficiently supported by preliminary or heterogeneous evidence. Further studies should prioritize standardized definitions of diminished ovarian reserve and premature ovarian insufficiency, well-designed prospective cohorts, randomized controlled trials, live birth and long-term health outcomes, and careful safety monitoring.

